# The empowering influence of air-liquid interface culture on skin organoid hair follicle development

**DOI:** 10.1093/burnst/tkae070

**Published:** 2025-01-16

**Authors:** Jane Sun, Imaan Ahmed, Jason Brown, Kiarash Khosrotehrani, Abbas Shafiee

**Affiliations:** Frazer Institute, Faculty of Medicine, The University of Queensland, Brisbane, QLD, 4102 Australia; Frazer Institute, Faculty of Medicine, The University of Queensland, Brisbane, QLD, 4102 Australia; Herston Biofabrication Institute, Metro North Hospital and Health Service, Queensland Health, Brisbane, QLD, 4029 Australia; Royal Brisbane and Women's Hospital, Metro North Hospital and Health Service, Queensland Health, Brisbane, QLD, 4029 Australia; Frazer Institute, Faculty of Medicine, The University of Queensland, Brisbane, QLD, 4102 Australia; Frazer Institute, Faculty of Medicine, The University of Queensland, Brisbane, QLD, 4102 Australia; Herston Biofabrication Institute, Metro North Hospital and Health Service, Queensland Health, Brisbane, QLD, 4029 Australia; Royal Brisbane and Women's Hospital, Metro North Hospital and Health Service, Queensland Health, Brisbane, QLD, 4029 Australia

**Keywords:** Air-liquid interface, Dermal papilla, Pluripotent stem cell, Regenerative medicine, Skin appendages

## Abstract

**Background:**

Rodent models have been widely used to investigate skin development, but do not account for significant differences in composition compared to human skin. On the other hand, two-dimensional and three-dimensional engineered skin models still lack the complex features of human skin such as appendages and pigmentation. Recently, hair follicle containing skin organoids (SKOs) with a stratified epidermis, and dermis layer have been generated as floating spheres from human-induced pluripotent stem cells (hiPSCs).

**Methods:**

The current study aims to investigate the generation of hiPSCs-derived SKOs using an air-liquid interface (ALI) model on transwell membranes (T-SKOs) and compares their development with conventional floating culture in low-attachment plates (F-SKOs).

**Results:**

Mature SKOs containing an epidermis, dermis, and appendages are created in both T-SKO and F-SKO conditions. It was found that the hair follicles are smaller and shorter in the F-SKO compared with T-SKOs. Additionally, the ALI conditions contribute to enhanced hair follicle numbers than conventional floating culture.

**Conclusions:**

Together, this study demonstrates the significant influence of transwell culture on the morphogenesis of hair follicles within SKOs and highlights the potential for refinement of skin model engineering for advancing dermatology and skin research.

Highlights

- Generation of human-induced pluripotent stem cells-derived skin organoids is optimized using an air-liquid interface model and compared with the conventional floating culture in low-attachment plates.
- Air-liquid interface culture advances the development of mature skin organoids with improved morphogenesis compared to floating culture.
- Air-liquid interface culture produces skin organoids with increased hair follicle numbers than floating culture.
- This study offers new insights into optimizing skin organoid models to enhance hair follicle development and dermatological research.

## Background

The generation of physiologically relevant human skin models is an ongoing research endeavor spanning many years. An accurate *in vitro* skin model would recapitulate the architecture and function of the human skin, serving as a valuable platform for investigating development, and disease progression. Furthermore, human skin models are necessary to reduce the reliance on animal models such as mice and pigs, where the skin morphology and disease processes have critical differences from that of human skin, complicating research translation [1, 2].

Classical *in vitro* methods of modeling human skin involve independently culturing specific skin cell types, such as keratinocytes and fibroblasts, before combining these into three-dimensional constructs resembling the layered structure of human skin [3–6]. However, these bilayer ‘skin equivalent’ models have limited functional and anatomical relevance due to their simplicity, rarely resulting in the formation of mature structures such as skin appendages, which are crucial for human skin function [7]. Therefore, the development of the human skin organoid (SKO) by Lee *et al*. in 2020 was a significant breakthrough in the field, as this skin model has far greater complexity and physiological accuracy than its predecessors [8].

Organoids are three-dimensional cultures that can be generated from human induced pluripotent stem cells (hiPSCs), a cell population with the potential to differentiate into any cell type or tissue of the human body. During the process of organoid generation, hiPSCs are guided by the modulation of specific developmental pathways, to direct their differentiation into the desired organ tissues. In SKO differentiation, for example, the modulation of the transforming growth factor beta (TGFβ) and fibroblast growth factor (FGF) signaling pathways co-induces the differentiation of epithelial and mesenchymal lineages, mimicking human fetal skin development [8, 9]. The co-induction and development of these populations is a major improvement to previous skin models, as it allows for epithelial-mesenchymal crosstalk, which is necessary to induce the formation of complex skin structures, in particular the pilosebaceous apparatus [10]. Hair follicles (HFs) are a key example of an appendage that relies on epithelial-mesenchymal crosstalk to develop and are therefore usually missing in other 2D or 3D skin culture systems [7].

In early gestation, HF patterning is initiated by signals from the mesenchyme, in conjunction with epithelial Wnt signaling, to induce the formation of thickened regions of epithelium, known as hair placodes [11, 12]. The hair placode epithelium invaginates into the dermis while signaling to surrounding mesenchymal cells to cluster and form the early dermal papilla. Further crosstalk between the epithelium and the early dermal papilla drives additional invagination and maturation, eventually leading to the development of a mature HF with sebaceous glands, inner and outer root sheath regions, and a hair shaft [7, 10]. The process of SKO generation, mimics embryogenesis, co-inducing the development of epithelial and mesenchymal populations, and thereby facilitating their natural interaction to form HFs [13]. This has established SKOs as a new platform to study skin and HF development, investigate hair loss disorders, and potentially apply for HF regeneration.

The pioneering protocol for human skin organoid generation, by Lee *et al*., involves forming hiPSC aggregates, maintaining these for 2 days in maintenance media, and then performing stepwise modulation of the TGFβ/FGF pathways to drive skin development [8]. Recently, an optimized SKO differentiation protocol was established by using direct embryoid body formation from hiPSCs as an immediate differentiation step, resulting in the formation of cystic SKOs that develop sensory neural networks, HFs, and sweat gland-like structures [9].

The air-liquid interface (ALI) model using the transwell system involves the use of a permeable membrane, where one side of the membrane is exposed to the surrounding air and the other side to the liquid media [14]. ALI has previously been used for culturing other human organoids, such as cerebral organoids, where it is demonstrated to not only improve organoid survival but also organoid functional development [15]. Similarly, when human SKOs were planarized and transferred to an ALI at Day 85 to 100 of culture, the maturation of the SKO epidermal layers was enhanced, as indicated by significantly increased stratum corneum formation and expression of mature keratinocyte markers, loricrin, and filaggrin [16]. This differentiation effect is expected, as exposure to air is known to promote the maturation and development of cultured epithelial cells, such as keratinocytes [14]. However, it remains to be determined what effect ALI culture has on SKO at early developmental timepoints, as opposed to late in the culture process when they have matured.

We believe that early intervention is a key factor in the development of hiPSC-derived organoids, particularly SKOs [9]. The changes that are applied at the late stages of SKOs development may have limited impact on improving skin structure. Therefore, introducing the ALI conditions immediately after cyst formation and before the development of skin appendages is essential to enhance SKO quality. Here, we investigated the effect of transferring SKOs generated using the optimized differentiation protocol [9] to ALI conditions on Day 12 of culture, to determine if ALI conditions were able to further improve SKO development.

## Methods

### Generation of hair-bearing human skin organoids

*hiPSC* culture: Three hiPSC lines, referred to as C32, P111, and P112, were used to generate hair-bearing SKOs (Supplemental Figure 1). The C32 hiPSC line was generated by reprogramming normal skin fibroblasts using the CytoTune 2.0 Sendai Reprogramming Kit (Thermo Fisher, Australia). The P111 and P112 hiPSC lines were generated with a similar method, using CD34+ cells isolated from human term placenta from two different individuals. The protocol was approved by the institutional human research ethics committee (HREC/09/QRBW/14). To isolate CD34+ cells, the placental tissues were minced and digested as previously described [17]. The single-cell mixture was subjected to Magnetic-Activated Cell Sorting (MACS) using CD34 MicroBead Kit (130–046-702, Miltenyi Biotec, Germany) to obtain CD34+ cells. CD34+ cells were cultured in StemPro-34 medium (Thermo Fisher Scientific, USA) supplemented with recombinant human FLT3 ligand (100 ng/ml, Peprotech, USA), SCF (100 ng/ml, Peprotech) and TPO (100 ng/ml, Peprotech). The CD34+ cells were expanded and used for iPSC reprogramming using CytoTune-iPS 2.0 Sendai Reprogramming Kit. The reprogramming was performed in mTeSR Plus medium (STEMCELL Technologies, catalog number 05850) on Matrigel-coated plates using human embryonic stem cells-(hESC) qualified Matrigel (Corning, catalog number 354277) at 37°C with 5% CO2. In the following weeks, the hiPSC colonies were identified by their compact shape, defined borders, and high nucleus-to-cytoplasm ratio, and were manually selected.

For expansion, the hiPSC lines were cultured in 6-well plates coated with 1% hESC-Qualified Matrigel and maintained in mTeSR medium supplemented with 0.5% antibiotic-antimycotic solution (Gibco, catalog number 15240096). The culture medium was changed daily or every other day. The cells were passaged at approximately 80% confluency, which is every 3 to 4 days [9].

*Differentiation of hiPSCs to SKOs*: Human SKOs were generated as previously described [18]. The three hiPSC lines were subcultured independently in 6-well plates coated with 1% Growth Factor Reduced (GFR) Matrigel (Corning, catalog number 354230). When the hiPSCs had reached 70–80% confluency, the cells from each hiPSC line were detached from the 6-well plate using Accutase (Sigma-Aldrich, catalog number A6964, 400–600 units/mL) and resuspended in mTeSR medium as a single cell suspension. The hiPSCs were further resuspended and then distributed at 8000 cells per 100 μL per well into a 96-well low attachment U-bottom plate in Essential 6 (E6) medium (Gibco, catalog number A1516401) supplemented with 0.5% antibiotic-antimycotic solution and 10 μM of Y-27632 (Rho-associated protein kinase (ROCK) inhibitor (STEMCELL Technologies)).

To aid in embryoid body formation, the plates were centrifuged at 250 g for five minutes and incubated in normal growth conditions overnight. This time point was designated as Day −1. The differentiation process was initiated at Day 0 when cells were exposed to 100 μl of fresh E6-based differentiation medium containing 2% GFR-Matrigel, 10 μM TGF-β signaling inhibitor (SB: SB43152, Miltenyi Biotec), 10 ng/mL BMP-4 (Lonza), and 4 ng/mL b-FGF (basic fibroblast growth factor, Miltenyi Biotec).

On Day 3, the formation of cranial neural crest cells (CNCC) was induced by adding low dose naltrexone (LDN, Lonza, catalog number 1066208-A) and b-FGF (Lonza, catalog number 100-18B) in a volume of 25 μL per well of the 96-well plate bringing the final volume of each well to 125 μL, with the final concentration of LDN and FGF2 being 1 μM and 250 ng/ml, respectively. On Day 6, the final volume in each well was brought to 200 μL with the addition of 75 μL of fresh E6 medium. On Days 8 and 10, 100 μL of the medium was removed from each well and replenished with 100 μL of fresh E6 medium. SKOs were randomly selected and divided into two groups, namely Floating SKO (F-SKO) and Transwell SKO (T-SKO).

**Figure 1:**
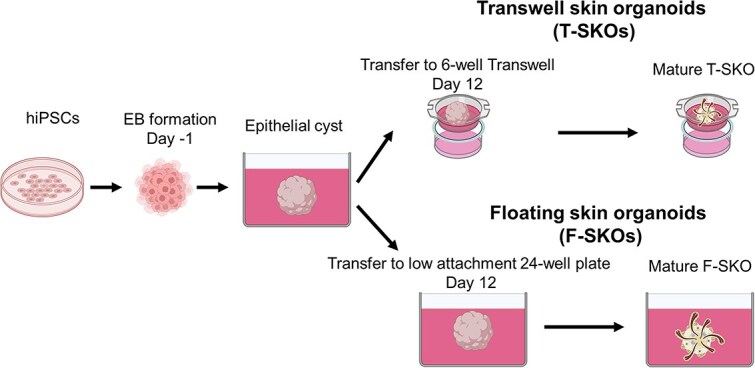
A schematic overview of the generation of skin organoids using human induced pluripotent stem cells subjected to sequential treatment with different molecules. For the air-liquid interface culture using the transwell membrane organoids (T-SKO) group, on day 12 of differentiation, the organoids were transferred on a 6-transwell membrane (24 mm diameter, 0.4 μm pore size), where three organoids were transferred onto an individual well with 1.5 ml of OMM, supplemented with 1% Matrigel. For the floating culture organoids (F-SKOs) group, on day 12 of differentiation, organoids were transferred into individual wells of a low attachment 24-well plate in 500 μl of organoid maturation medium (OMM), supplemented with 1% Matrigel, and continued culturing in floating conditions. Schematic images created with BioRender (www.biorender.com)

*Floating SKOs:* For the development of F-SKOs, on Day 12 of differentiation, the organoids, shaped as epithelial cysts, were transferred into individual wells of a low attachment 24-well plate in 500 μL of organoid maturation medium (OMM), supplemented with 1% Matrigel (Figure 1**,** and Supplemental Figure 2). On Day 15, a half-medium change was performed with OMM supplemented with 1% GFR-Matrigel. On Day 18, a half-medium change was performed with OMM only. Half medium changes were then performed every other day until Day 130 of culture, using OMM only. The media volume was increased to 1.2 mL per well of the 24-well plate from Day 80 onwards, as the SKO matured and grew larger. All differentiation mediums and OMM contained 1% antibiotic-antimycotic.

*Transwell SKO*: To establish the ALI culture system for skin organoids (T-SKOs), organoids (at Day 12 of differentiation, and when cyst formation was confirmed) were transferred onto a 6-well transwell membrane (24 mm diameter, 0.4 μm pore size) to facilitate optimal nutrient exchange and structural support. (Figure 1**,** and Supplemental Figure 2). Three of Day 12 organoids were transferred onto the apical side of an individual Transwell insert in 1.2 ml of OMM supplemented with 1% GFR-Matrigel and maintained in a humidified incubator at 37°C with 5% CO_2_. The transfer was performed using a 1000 μL pipette tip with a wide bore to ensure precise placement and minimize mechanical stress on the organoids. The OMM was positioned beneath the membrane such that only the basal surface of the organoids had contact with the medium and the apical surface of organoids interfaced with the air. This setup recapitulates the physiological and anatomical conditions of the skin, while the outer layer is exposed to air, the dermis is connected and nourished by underlying tissues. Throughout the differentiation period, the medium on the basal side was refreshed.

On Day 15, the media was replaced with OMM (600 μL) containing 1% GFR-Matrigel. From Day 18 until the endpoint of the experiment, medium change was performed every other day with fresh OMM with 1x antibiotic-antimycotic and without Matrigel for T-SKOs, like F-SKOs. However, full medium changes were performed for T-SKOs, while half medium changes were performed for F-SKOs. As the organoids matured and grew larger, the volume of the medium was increased to 1.5 mL per well of the 6-well plate for T-SKOs.

### Bright field microscopy

Bright-field (BF) images of organoids were taken at different timepoints using an Olympus IX73 inverted microscope. Five organoids were randomly chosen for brightfield imaging from each hiPSC line at Day 0. BF images of these same organoids were also taken at other specific timepoints. BF images from Days 65, 75, 85, 95, and 115 of SKO differentiation are presented in this study.

#### Immunofluorescence and histology analyses

Immunostaining analyses were performed on SKOs at Days 16, 50, 65, 75, 85, 90, and 120 of culture. An average of five SKOs were randomly selected from each group at each timepoint and fixed in 4% paraformaldehyde (PFA). The fixed SKOs were embedded in cryomolds with Optimal Cutting Temperature (OCT) cryo-embedding medium and stored at −20°C for cryo-sectioning. Each embedded sample was sectioned into 20 μm and 50 μm thicknesses using the Leica Cryostat CM1950 cryostat machine and then transferred to Superfrost Plus slides (ThermoFisher, catalog number J7800AMNT). The sectioned samples were then stored in the −20°C freezer until ready to be used for immunostaining.

For immunostaining, cryosections were blocked with blocking buffer (1xPBS/10% goat serum/0.3%Triton X-100, from Sigma-Aldrich, catalog number NS02L) for 10 minutes at room temperature (RT), and then incubated with primary antibodies at 4°C overnight. Supplemental Table 1 shows the list of antibodies used in the current study. The primary antibodies were removed the following day, and the samples were washed thrice with washing buffer (1x PBS + 0.1% Trixton 100 solution) before incubation with secondary antibodies for at least an hour at RT in the dark. Then, samples were washed twice with washing buffer and cell nuclei were stained with 4′,6-diamidino-2-phenylindole (DAPI, DAKO, catalog number G9406) for 10 minutes in the dark at RT. Samples were imaged using a Nikon/Spectral Spinning Disc Confocal Microscope.

To perform whole-mount immunofluorescence analysis, first, SKOs were fixed in 4% PFA in PBS. Whole SKOs were blocked with blocking buffer at RT for 3 hours, before incubating with primary antibodies for 2 days at 4°C. Organoids were then washed 3 times with washing buffer, 30 minutes each, before incubating with secondary antibodies at RT for 4 hours or at 4°C overnight. Organoids were then washed 3 times and stained with DAPI at RT for 1 hour. Organoids were then transferred into RapiClear (Sigma) at RT until they were cleared, usually overnight. Immunofluorescence images from whole-mount organoids were taken using a Nikon/Spectral Spinning Disc confocal.

Organoid histology was assessed with Hematoxylin and Eosin (H&E) staining. The organoids were fixed in 4% PFA and then washed with PBS and incubated in 70% (v/v) ethanol until further analysis. Specimens were dehydrated with a graded ethanol series and embedded in paraffin. Next, at least 30 continuous 6 μm thick tissue sections were obtained and stained with H&E stain. High-resolution images were acquired using a slide scanner (VS200, Olympus, Japan). Images were analyzed using the ‘Olympus Olyvia’ software.

#### Statistical analysis

All data were analyzed using an unpaired two-way ANOVA with Multiple Comparisons (Tukey’s multiple comparisons test) using GraphPad Prism (version 9.2.0). Quantitative data was presented as mean ± standard deviation (SD). The *P* value < 0.05 was considered statistically significant. For immunofluorescence analysis and histology, at least three biological replicates were prepared and analyzed.

## Results

### ALI culture results in accelerated development of skin organoid

To examine the differences in SKO development and maturation between the ALI and floating culture conditions, we established the two-culture systems as described in Figure 1. The SKO differentiation procedure, from Day −1 to Day 12, was the same for both culture systems and was performed as previously described in Shafiee *et al*. [9]. On Day 12 of differentiation, the SKOs were randomly divided between the two culture conditions (ALI or floating) (Figure 1).

**Figure 2:**
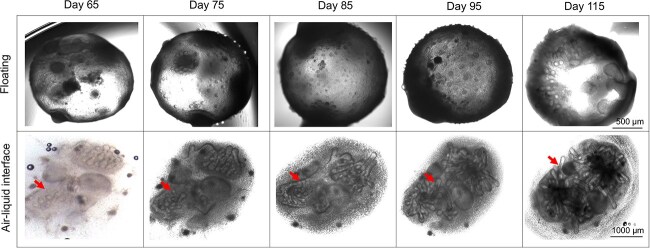
Air–liquid interface culture using the transwell system contributes to the development of enhanced hair follicles (HF) quantity and length in human induced pluripotent stem cells derived-skin organoids (SKOs). Representative brightfield images of C32-derived SKOs using the floating (top row, scale bar: 500 μm) and air-liquid interface with transwell (bottom row, scale bar: 1000 μm) differentiation conditions for up to 115 days. Red arrows represent the differentiation of a single hair follicle in the same SKO over time

Human primary tissue organoids range in size from a few hundred micrometers to a few millimeters (mm) and do not grow beyond a few millimeters in culture. Nevertheless, the small size of current organoids remains a limiting factor that restricts their ability to fully recapitulate the late stages of human organ development [19]. Here, floating SKOs varied in size, ranging from 1 mm to a maximum of 3 mm in diameter by day 115. The transwell culture resulted in the development of SKOs with similar sizes, and T-SKOs were consistently larger than F-SKOs and could grow up to around 4 mm in size by day 115 (Figure 2). While the F-SKOs were spherical, the T-SKOs displayed a domed structure and became large and fused within the transwell.

The HF growth in T-SKOs and F-SKOs was monitored at designated intervals (Days 65, 75, 85, 95, and 115) and BF images were taken from individual SKOs and analyzed. The BF images displayed the development of visible hair placodes at Day 65 in both groups (Figure 2, representative BF images of C32-derived SKOs differentiated using the floating (top row) and ALI (bottom row) culture systems, images taken from the same SKOs over time). In the following timepoints, the number of hair placodes increased, leading to their transition into HFs. The transwell cultures allow for longitudinal observation of individual HF growth over time, while the floating SKOs undergo rotational movement making the continuous tracking impractical. Compared with the floating system, HFs exhibited greater elongation in transwell culture over time, reaching a length of up to 1 mm by Day 115 (Figure 2). Additionally, the HFs in T-SKOs exhibit features suggestive of hair shafts (dark and elongated structure), resembling the characteristics of HF development (Figure 2, images from Day 95 and Day 115). Generation of advanced-stage HFs with extended length enables detailed investigation of the composition, development, and structural integrity of these follicles.

**Figure 3:**
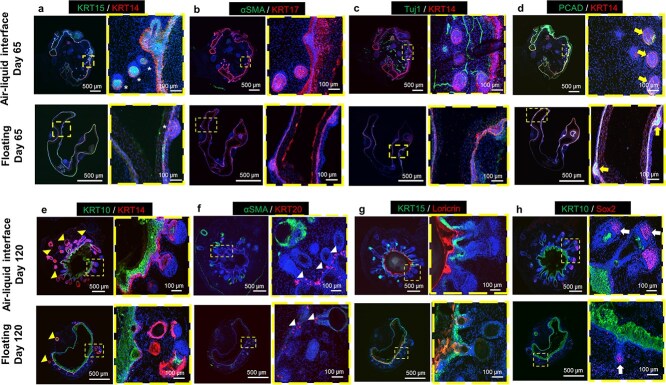
Culture in the air-liquid interface (ALI) differentiation conditions enhance the development of human induced pluripotent stem cell-derived skin organoids (SKOs). (**a**-**d**) Representative immunostaining images of sectioned C32-derived SKOs in ALI (top) or the floating (bottom) differentiation conditions at day 65. Dashed yellow boxes indicate the magnified regions. White asterisks in part a and yellow arrows in part D represent hair follicles (HFs). Keratin 15 (KRT15) and keratin 14 (KRT14) staining mark the epidermis basal layer and hair placodes. (b-d) Keratin 17 (KRT17) and KRT14 staining indicates proliferative keratinocytes and the HF outer root sheath. αSMA staining marks smooth muscle actin protein. Neuron-specific class III beta-tubulin (TUJ1) staining confirms the innervation of the dermal layer at day 65. (**e**-**h**) Representative immunostaining images of sectioned SKOs in ALI (top) or the floating (bottom) differentiation conditions at day 120. Dashed yellow boxes indicate the magnified regions. (e) Keratin 10 (KRT10) and KRT14 staining mark the differentiated keratinocytes, and epidermis basal layer, respectively. Yellow arrowheads in part E represent hair follicles (HFs). (f) Keratin 20 (KRT20) staining confirms the presence of Merkel cells in SKOs as indicated by white arrowheads. (g) KRT15 is expressed by the epithelium layer, and loricrin marks the terminally differentiated cornified keratinocytes. (h) SRY-box transcription factor 2 (Sox 2) staining indicates the HF dermal papilla as indicated by white arrows. Blue indicates cell nuclei stained with 4′,6-diamidino-2-phenylindole (DAPI). Scale bars are 500 μm for the whole organoids’ sections and 100 μm for the magnified sections

To evaluate the influence of transwell culture on SKO development, they were subjected to immunostaining using key skin protein markers, including KRT14, KRT15, αSMA, KRT17, TUJ1, PCAD, KRT10, KRT20, Loricrin, and SOX2 at Day 65 and Day 120 of SKO maturation (Figure 3**,** Representative immunostaining images of sectioned SKOs in ALI (top) or the floating (bottom) differentiation conditions at Day 65 and Day 120). The development of SKOs proceeded similarly in both conditions. The presence of HF placodes was confirmed within the SKOs by Day 65 (Figure 3a**)**. The hair placodes were observed in the interface of dermal and epidermal layers, as the result of epidermal thickening [20]. The HF placodes were observed within the dermal layer potentially due to their invagination into the dermis (Figure 3b). Immunostaining analysis confirmed the expression of K14, P-Cadherin, and K17 in the HF placodes. K17 was highly expressed at the HF placode and throughout the thickened epidermis (Figure 3b). Beta-tubulin (TUJ1) staining was used to assess the innervation of SKOs [8] and confirmed the innervation of SKOs by Day 65, which was more prominent in the T-SKO compared to F-SKO (Figure 3c).

**Figure 4:**
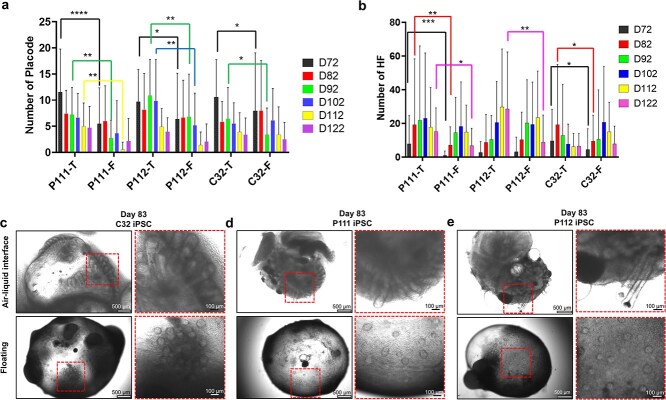
Culture in air-liquid interface (ALI) differentiation conditions using the transwell system contributes to an enhanced hair inductive capacity in human-induced pluripotent stem cell (iPSC)-derived skin organoids (SKOs). The graphs represent the quantitative data from SKOs differentiated in the floating (F, n = 72 SKOs) or on transwell (T, n = 60 SKOs) differentiation conditions. SKOs were observed under the microscope and assessed for protruding hair placodes (**a**) or hair follicles (HFs, (**b**) at different time points. Data from C32, P111, and P112 human induced pluripotent stem cell lines (iPSC) differentiated to SKO for up to 122 days. The C32 iPSC line was generated from normal skin fibroblasts, and the P111 and P112 iPSC lines from CD34+ cells isolated from two different term placenta. Data are mean ± standard deviation. Two-way ANOVA with multiple comparisons. ^*^p < 0.05, ^*^^*^p < 0.01, ^*^^*^^*^p < 0.001, ^*^^*^^*^^*^p < 0.0001 denotes significance. (**c**, **d**, and **e**) representative bright field images of SKO at day 83. Dashed red boxes indicate the magnified regions. Scale bars: 500 μm for the whole organoid, and 100 μm for the magnified regions

The SKOs underwent a transition from a thin bilayer structure at Day 65 (Figure 3a-3d) to a multilayer skin-like structure by Day 120 (Figure 3e-3h). Matured SKOs formed a multilayered epithelium with a high level of K10 expression in the outer (suprabasal) layer, whereas K14 expression was detected in the basal layer of the epidermis and the HFs regions (Figure 3e). K15 expression was also detected in the basal layer of the epidermis (Figure 3g). Loricrin, a key marker of terminally differentiated epidermal cells was much more robustly and regularly present in T-SKO as compared to F-SKO at Day 120 (Figure 3g).

For both culture conditions, hair placode induction occurred on Day 65, and HFs reached *in vitro* maturation by Day 120. The thickness of the dermal layer was greater in T-SKOs compared with the F-SKOs. SOX2 is a key protein in the dermal papilla for the development of HFs in mammals [21]. In SKOs, SOX2-positive cells were present immediately beneath the basal cell layer of the epidermis and HFs indicating their expression in dermal papilla cells (Figure 3h). Of note, the HFs in the T-SKOs exhibited increased length, and a higher number of SOX2-positive cells (Figure 3h**,** top). The results indicate that SKOs could be successfully generated from both culture systems, and that transwell culture promoted differentiation of epidermal layers and maturation of HFs. Immunostaining data confirmed an increase in the number and length of HFs in T-SKOs compared with F-SKOs (Figure 3e-3h).

**Figure 5:**
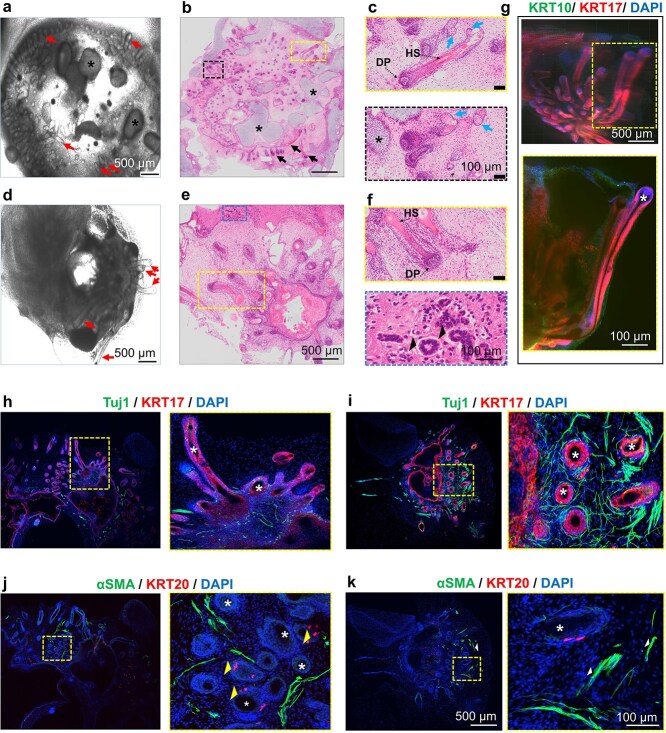
Human induced pluripotent stem cell (hiPSC)-derived skin organoids (SKOs) generate sweat and sebaceous glands when differentiated using air-liquid interface conditions. (**a**) Representative bright-field images of SKOs generated from the P112 hiPSC line on the transwell at day 90. Red arrows indicate the hair follicles (HFs), and black asterisks indicate the cartilaginous parts. (**b** and **c**) representative hematoxylin and eosin (H&E) staining images, from the same SKOs shown in part a, confirm the presence of hair shafts (HS, dashed arrows), dermal papilla (DP), and sebaceous glands (blue arrows). (**d**) Representative bright-field images of SKOs generated from the P112 hiPSC line on the transwell at day 120, red arrows indicate the HFs. (**e** and **f**) Representative H & E staining images, from SKOs confirm the presence of HS, DP (dashed arrows), and sweat glands (black arrowheads). (**g**-**k**) Representative immunostaining images of SKOs on transwell. Dashed yellow boxes indicate the magnified regions. (g), and (h) keratin 17 (KRT17) staining confirms the presence of HFs within the SKOs as indicated by white asterisks. (i) Neuron-specific class III beta-tubulin (TUJ1) staining confirms the innervation of the dermal layer at day 120. (j, and k) immunostaining analysis confirms the presence of smooth muscle actin (SMA) positive arrector pili muscles and keratin 20 (KRT20) positive Merkel cells next to the HFs. The blue color indicates cell nuclei stained with 4′,6-diamidino-2-phenylindole (DAPI). In parts i and j, the white asterisks, yellow arrowheads, and white arrowheads indicate HFs, Merkel cells, and arrector pili muscles respectively. Scale bars are 500 μm for the whole organoid sections in parts a, b, d, e, g, h, i, j, and k, and 100 μm for parts c, f, and inserts in parts g, h, i, j, and k

**Figure 6:**
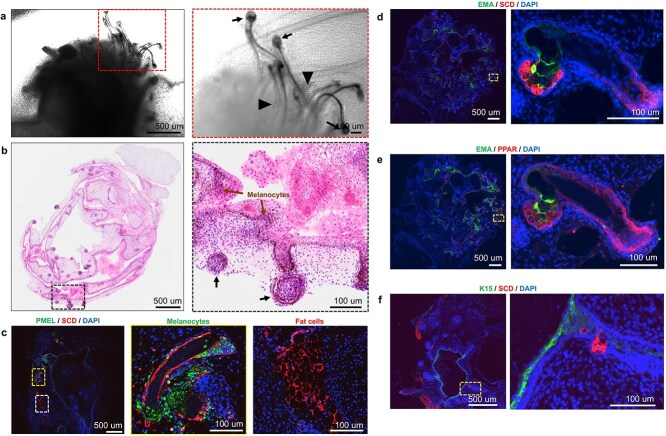
Human induced pluripotent stem cell (hiPSC)-derived skin organoids (SKOs) are pigmented when differentiated using a transwell system. (**a**) Representative bright-field images of SKOs generated from the P111 hiPSC line on the transwell at day 130. Black arrowheads indicate the fat tissue formation next to the hair follicles (HF, black arrows). (**b**) Representative hematoxylin and eosin (H&E) staining images confirm the presence of pigmented cells (brown arrows) in the epidermis and around the HFs (black arrows) within the SKOs. (**c**) the presence of melanocytes and fat tissue within SKOs was confirmed by immunoassaying for melanoma gp100 antibody (PMEL, green color) and anti-acyl-CoA desaturase protein (SCD, red color), respectively. d-f) Representative immunostaining images of SKOs on transwell. (**d**) Immunostaining analysis confirms the presence of SCD (red color) positive and epithelial membrane antigen (EMA, green color) positive sebaceous glands next to the HFs. (**e**, **f**) sebaceous glands are also positive for peroxisome proliferator-activated receptor (PPAR)-gamma (red color), but negative for cytokeratin-15 (K15, green color) marker. The blue color indicates cell nuclei stained with 4′,6-diamidino-2-phenylindole (DAPI). Dashed boxes indicate the magnified regions. Scale bars are 500 μm for the whole organoid sections in parts a, b, c, d, e, and f, and 100 μm for the magnified regions

The generation of hiPSCs-derived SKOs using the transwell membranes was investigated using 2 other cell lines, P111 and P112 hiPSC lines, both generated from CD34+ cells isolated from two different human term placentas. The representative IF images of P111 and P112 cell line-derived T-SKOs at Day 65 and Day 120 are shown in Supplemental Figure 3.

### Air-liquid interface culture improved generation of HF within skin organoid

The number of hair placodes and HFs within individual SKOs, as an indicator of SKO development and function, was counted at different time points (Days 72, 82, 92, 102, 112, and 122) (Figure 4). The hair placodes and HFs were defined as a circular cluster of cells and a three-dimensional bulbous structure that protruded from the SKO’s surface, respectively [9]. The hair placodes became discernible on SKOs under the microscope around Day 65 of differentiation and were quantifiable from Day 72, allowing quantification from this timepoint. It was found that both culture systems induced the formation of hair-bearing SKOs using the P111, P112, and C32 hiPSC lines (Figure 4a). The highest number of hair placodes was detected on Day 72 for both P111 and C32 hiPSC lines cultured in both transwell and floating conditions, reducing in the subsequent time points. On Days 72 and 92, a statistically significantly higher number of hair placodes was detected in the T-SKO group compared to the F-SKO across all three hiPSC lines (Figure 4a**,** P < 0.05). For both P111 and P112 hiPSC lines, the number of hair placodes was higher in T-SKOs compared to F-SKOs across all intervals (Figure 4a).

In the transwell group, the number of HFs increased from Day 72 onward and peaked on Day 102, Day 112, and Day 82 for P111, P112, and C32 hiPSC lines, respectively (Figure 4b). P111 hiPSC-derived T-SKOs consistently showed a higher number of HFs compared to F-SKOs at all time intervals. Similar results were observed for P112 hiPSC-derived T-SKOs on Days 112 and 122. Transwell culture induced HF production at an earlier timepoint in the C32 hiPSC line. On Days 72, 82, and 92, the C32 T-SKOs showed a higher quantity of HFs, whereas the C32 F-SKOs exhibited greater numbers on Days 102, and 112 (Figure 4b). Figure 4c, 4d, and4e show the representative bright field images of different SKO at day 83 in both transwell and floating culture conditions. In summary, both Transwell and floating culture conditions support the generation of HF-bearing SKOs across multiple hiPSC lines, with the transwell culture demonstrating enhanced efficacy in HF development and maturation. Additionally, the HF generation potential varied based on the hiPSC line.

### Multilineage differentiation and innervation of skin organoids

*De novo* generation of skin substitutes with specialized cell types poses a significant challenge in the field [7]. We therefore examined the different cell types or structures that could be identified within skin organoids in transwell culture conditions. Figure 5a shows a bright-field image of hair-containing T-SKOs from the P112 hiPSC line at Day 90. H&E staining confirmed an abundant of HFs within the T-SKO (Figure 5b). Dense cartilaginous regions were identified within the SKOs (Figure 5a, black asterisks) indicating the need for further optimization of differentiation protocols to prevent off-target (chondral) tissue development. By Day 90, areas of lipid-rich sebaceous glands were detected alongside the HFs (Figure 5c, blue arrows). The HF length was increased over time, with HFs protruding from the organoids by Day 120 (Figure 5d**,** red arrows). H&E staining showed several tubular-shaped structures within the SKOs suggesting the development of eccrine sweat glands by Day 120 (Figure 5e and5f, black arrowheads). Figure 5g shows representative immunostaining images of SKOs on the transwell, and Keratin 17 (KRT17) staining confirms the presence of HFs within the SKOs (white asterisks).

Immunostaining with neuron-specific class III beta-tubulin (TUJ1) validated the innervation of the T-SKO dermal layer on Day 120 (Figure 5h). Neural networks, represented by muti-neural fascicules, were evident around the HFs (Figure 5i, representative immunostaining images of T-SKOs at Day 120, KRT17+ staining confirming the presence of HFs within the T-SKOs marked by white asterisks). In addition, immunostaining confirmed the presence of smooth muscle actin (SMA) positive cells linking hair follicles to the surrounding epidermis, indicative of arrector pili muscles. KRT20-positive cells were suggestive of Merkel cells adjacent to HFs (**Figure 5j**, and**5k****,** white asterisks indicating the HFs). However, the production of arrector pili muscles and Merkel cells within the SKOs requires further investigation, as these results are indicative and preliminary. Supplementary Video S1, S2, andS3 show a representative 3D reconstruction of T-SKOs, stained with keratin 10 (KRT10, green color), and keratin 17 (KRT17, red color) antibodies, at Day 90, Day 120, and Day 130 of differentiation, respectively.

Moreover, the bright-field imaging confirmed the generation of pigmented T-SKOs in transwell culture systems on Day 130 (Figure 6a**,** as indicated by black arrows). The pigmented HFs exhibit a dark coloration and protrude from the T-SKOs (Figure 6a). Our investigation revealed the presence of adipose tissue near the HFs (Figure 6a**,** as indicated by black arrowheads), suggesting a critical contribution to the regulation of HFs cycling [22]. Notably, the T-SKOs exhibited longer HF lengths compared to those in F-SKOs (Supplemental Figure 4) and resembled the natural process of HF development *in vivo* [23, 24]. H&E staining showed brown pigment coloration in the epidermis and around the HFs indicating the development of melanocytes within T-SKOs (Figure 6b). Melanoma gp100 antibody (PMEL) positive immunostaining further confirmed the presence of melanocytes within SKOs (Figure 6c).

The development of hypodermis within the SKOs has been a major challenge. The Hypodermis layer not only contributes to the mechanical and thermoregulatory properties of the normal skin but also supports fibroblast proliferation during skin wound healing [22, 25]. Here, a significant number of adipocytes within the hypodermis was detected within (Figure 6c) and at the outermost layer (Figure 6a, and Figure 6c) of T-SKOs confirmed by immunoassaying for anti-Acyl-CoA desaturase protein (SCD, red color, Figure 6c). Immunostaining analysis confirmed the presence of SCD positive, and Anti-Epithelial membrane antigen (EMA) positive sebaceous glands next to the HFs (Figure 6d). Sebaceous glands were positive for Peroxisome proliferator-activated receptor (PPAR)-gamma, but negative for cytokeratin-15 (K15) marker (Figure 6e, 6f).

## Discussion

To date, several approaches have been employed to create human skin models *in vitro*. In the current study, the generation of hiPSC-derived SKOs was evaluated, where organoids were developed on commercial transwell inserts at an air-liquid interface (ALI culture) and compared with organoids established using the classical floating culture conditions.

ALI culture has been widely employed for developing hiPSC-derived organoids, such as cerebral organoids, kidney organoids, and intestinal organoids [15, 26, 27]. These previous studies have reported enhanced maturation, development, and function of human organoids in the laboratory [16, 28]. Similarly, the use of the air-liquid interface system resulted in enhanced epidermal stratification and differentiation in human-skin-equivalent cultures [29]. Additionally, culturing on ALI has been suspected to improve HF maturation [16].

In the current study, SKOs were cultured on transwell membranes at Day 12 of differentiation. We hypothesized that the early induction of SKOs in a transwell system minimizes cyst formation and enhances the formation of skin structures. The histology analyses demonstrated improved HF induction, development, and morphogenesis in the ALI system compared with the classical floating culture method. Furthermore, the ALI culture did not alter the development of skin layers (epidermis, dermis, and hypodermis areas), reduced the variation between individual SKOs, and provided more clinically relevant material for transplantation or sample collection. Notably, the use of the ALI system significantly increased the quantity of HFs within SKOs compared to floating culture.

In all the examined cell lines, the number of hair placodes was consistently higher in ALI culture across most timepoints. Human HFs with arrector pili muscle contraction, play an important role in body temperature regulation [30]. Lee *et al*. reported that arrector pili muscle-like features were extremely rarely observed in their culture condition, indicating that the SKO is still at an early developmental stage and requires further optimization of the culture condition to speed up the maturation [8]. Arrector pili muscle-like features were frequently observed in T-SKOs at Day 120 (Figure 5j, 5k), but were rarely observed in F-SKOs, suggesting that the ALI condition is helpful for the induction and maturation of arrector pili muscles.

Although the precise mechanisms by which the ALI system enhances HF generation remain to be understood, several factors may contribute to this outcome. ALI culture provides a biomimetic microenvironment mimicking the skin's physiological condition. The ALI system offers a supporting scaffold that promotes growth, differentiation, and stratification of keratinocytes while facilitating epithelial-mesenchymal interactions critical for HF morphogenesis. Moreover, the ALI culture may influence the key signaling pathways, such as Wnt/β-catenin, and BMP, which are important for HF development. Further investigations are required to understand the role of mechanical and biochemical cues in ALI culture in promoting skin organoid development and HF generation.

The presence of HFs within engineered grafts could not only provides better cosmetic outcomes but also improves skin graft take rate [31]. Plotczyk M *et al.* showed that transplantation of HF-containing transplants contributes to faster healing and less scarring as compared to non-HFs-containing transplants [32]. HFs induce a long-term systematic change to the transplantation sites [32], as the dermal population of HFs contributes to dermal remodeling, while the epithelial stem cells within the HFs migrate, and support wound re-epithelialization [33]. Although, previous tissue-engineered constructs have shown promising outcomes in large full-thickness skin defects, preventing dehydration and infection, ultimately, they are limited in the generation of skin appendages [7]. Therefore, accessing HF-containing skin substitutes could potentially enhance skin regeneration post-trauma. *In vivo* assessment of the regenerative capacity of HF-containing organoids remains to be investigated in future studies.

Additionally, the generation of advanced-stage HFs with extended length enables detailed investigation of composition, development, and structural integrity providing insights into new skin therapeutics or cosmetics. Successfully achieving *in vitro* pigmented skin models would also provide a research platform to advance investigations into pigment-related skin disorders and devise new strategies for restoring hair pigmentation.

While the use of an ALI system contributed to improved skin structure and generation of abundant HFs, the off-target (cartilage) tissue formation persisted within the SKOs. The presence of cartilaginous regions within the SKOs highlights the necessity for further optimization of differentiation protocols to mitigate off-target differentiation [18]. Additionally, the SKOs still lack other cell populations, including blood vessels and immune cells, which limits the size and maturation of HFs within the SKOs. Vascularization is also essential for long-term culture and the application of HF-containing organoids for wound healing and transplantation studies. Therefore, future investigations should prioritize the induction of vascular and immune systems within SKOs.

Human sweat glands, especially their progenitors during embryonic development, remain elusive due to ethical constraints. Previous studies on skin organoids found that these glands are immature [9]. Therefore, further research is needed to find improved methods for identifying and analyzing fetal sweat gland progenitors.

The absence of critical cell populations, including blood vessels and immune cells, and the prolonged culture duration to obtain mature SKOs highlight the necessity for ongoing refinements of differentiation protocols to achieve a more precise human skin equivalent. In addition, the extended *in vitro* culture time exceeding 4 months for the generation of SKOs poses another limitation for their potential translational and clinical applications, necessitating careful consideration and resolution in the future. Stabell *et al.* reported that hypoxic culture can rescue some aberrant signaling programs in human-skin-equivalent organoids and mimic physiological levels, thus improving the maturation of skin equivalents [34]. The implementation of hypoxic conditions may accelerate the maturation of HF-containing SKOs.

## Conclusions

 Human skin tissues cultured under ALI conditions acquired terminal differentiation, highlighting the critical role of the ALI system in promoting skin maturation. Additionally, ALI culture conditions have promoted human organoid differentiation. The current research leveraged the transwell model to recapitulate an ALI condition, as a critical strategy for SKO development, and demonstrated the efficacy of ALI culture using transwell membranes in enhancing the development and maturation of hiPSC-derived SKOs. We found that using ALI culture at this early timepoint resulted in enhanced HF production, length, and morphology, creating an improved platform for the study of skin development, and opening new avenues for HF regeneration. The implementation of the ALI technique resulted in improved epidermal stratification, HF induction, and morphogenesis compared to classical floating culture methods. The adoption of the ALI conditions facilitated the formation of larger skin tissues and presented reduced variation between individual SKOs, offering clinically relevant materials for tissue analyses and transplantation trials. The engraftment of HF-containing skin has shown faster wound healing and less scarring. Therefore, the increased number of HFs within SKOs in ALI conditions may improve the skin graft take rates and contribute to better cosmetic outcomes. Overall, the findings presented here contribute valuable insights into the pursuit of developing patient-specific complex skin analogs for both research and therapeutic purposes. These insights pave the way for future studies aimed at improving skin regeneration and treating skin disorders.

## Supplementary Material

Supplemental_Materials_3rd_Revision_tkae070

## Data Availability

The data that support the findings of this study are available from the corresponding author upon reasonable request.
