# Supplementary material for: The empowering influence of air-liquid interface culture on skin organoid hair follicle development
Source: Burns Trauma. 2025 Jan 16;13:tkae070. doi: 10.1093/burnst/tkae070 (PMC11736897; doi:10.1093/burnst/tkae070)
Supplement: Supplemental_Materials_3rd_Revision_tkae070 [file supplemental_materials_3rd_revision_tkae070.docx]

**The Empowering Influence of Air-Liquid Interface Culture on Skin Organoid Hair Follicle Development**

**Supplementary Information**

***Supplementary Table 1:*** *Antibodies used in this study for immunofluorescence analysis.*

| **Antibody name** | **Source species** | **Company** | **Catalog number** | **Dilution** |
| --- | --- | --- | --- | --- |
| Anti-alpha smooth muscle actin monoclonal antibody | Mouse | Abcam | ab7817 | 1:100 |
| Anti-beta III tubulin (Tuj1) polyclonal antibody | Mouse | BioLegend | 801202 | 1:200 |
| Anti-collagen 2A1 Monoclonal Antibody | Mouse | ThermoFisher | MA1-37493 | 1:200 |
| Anti-cytokeratin-10 monoclonal antibody | Mouse | Abcam | ab9026 | 1:200 |
| Anti-cytokeratin-14 monoclonal antibody | Rabbit | Abcam | ab181595 | 1:100 |
| Anti-cytokeratin-17 monoclonal antibody | Rabbit | Abcam | ab109725 | 1:100 |
| Anti-cytokeratin-20 monoclonal antibody | Rabbit | Cell Signaling Technology | 13063S | 1:100 |
| Anti-E-cadherin monoclonal antibody | Mouse | BD Biosciences | 610181 | 1:100 |
| Anti-loricrin monoclonal antibody | Rabbit | Abcam | ab198994 | 1:100 |
| Anti-P-cadherin monoclonal antibody | Mouse | Invitrogen | 32-4000 | 1:100 |
| Anti-SOX2 monoclonal antibody | Rabbit | Cell Signaling Technology | 3579 | 1:50 |
| Anti-stearoyl-CoA desaturase (SCD) | Rabbit | Sigma | HPA012107 | 1:200 |
| Anti-premelanosome protein (PMEL) | Mouse | Merck | MABC1720 | 1:100 |
| Anti-epithelial membrane antigen (EMA) | Mouse | Agilent | M061329-2 | 1:100 |
| Anti-peroxisome proliferator-activated receptor (PPAR)-gamma | Rabbit | Abcam | ab59256 | 1:100 |
| Anti-cytokeratin-15 monoclonal antibody | Mouse | GeneTex | GTX72325 | 1:100 |
| IgG (H+L) Highly Cross-Adsorbed Secondary Antibody, Alexa Fluor 568 | Donkey anti-rabbit | Life Technologies Corporation | A10042 | 1:500 |
| IgG (H+L) Highly Cross-Adsorbed Secondary Antibody, Alexa Fluor 488 | Goat anti-mouse | Life Technologies Corporation | A11029 | 1:500 |


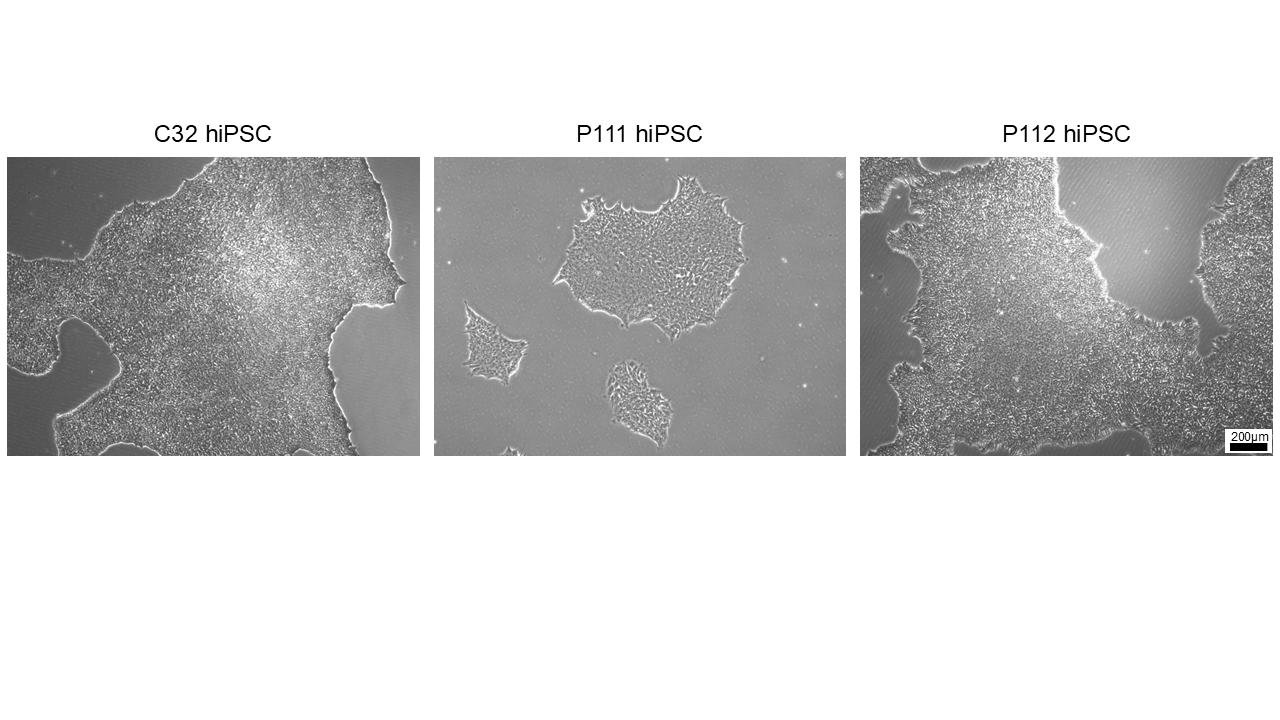


***Supplementary Figure 1:*** *Representative bright filed images of C32, P111, and P112 human induced pluripotent stem cells (hiPSC). Scale bars are 200 μm.*


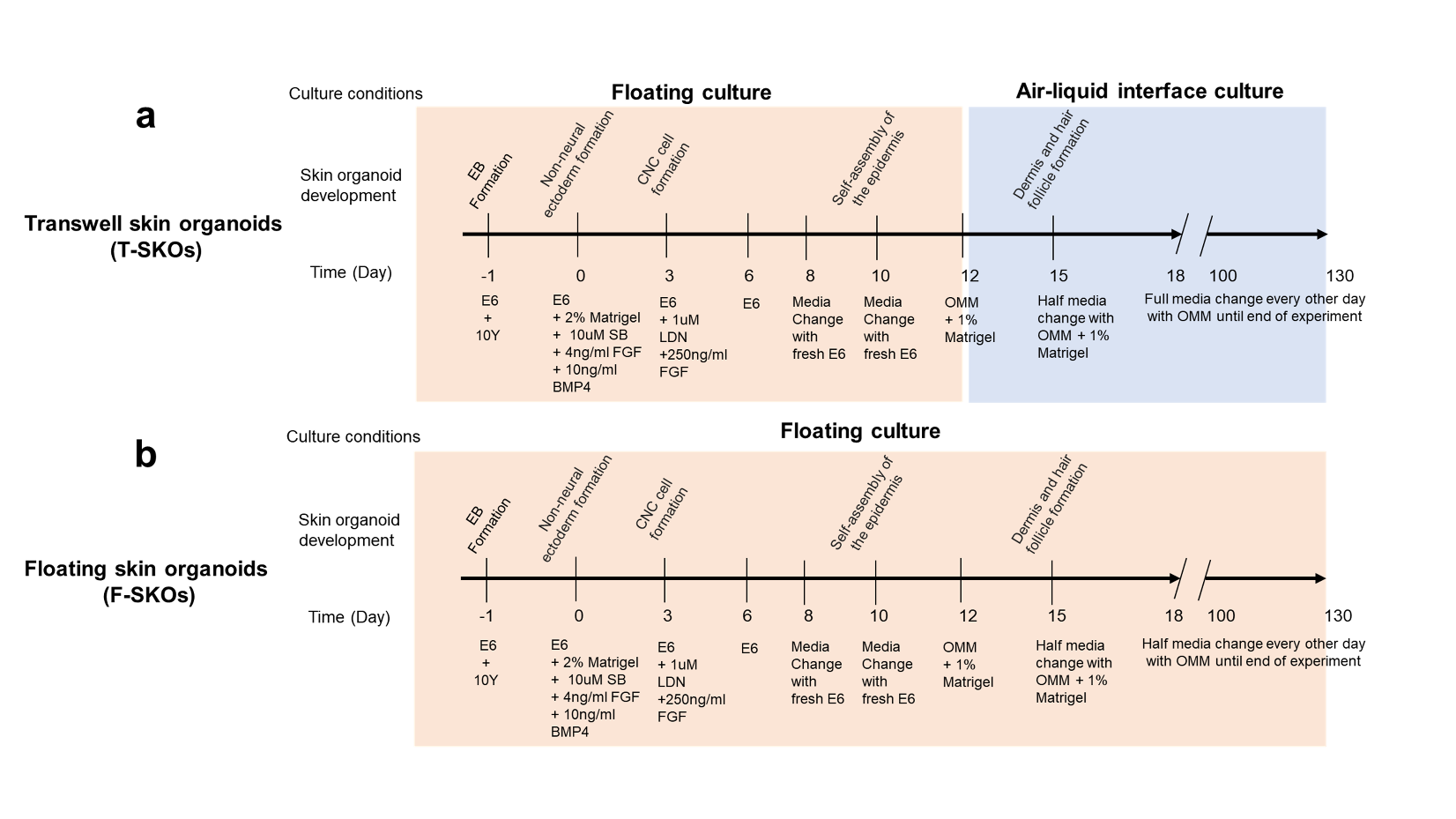


***Supplementary Figure 2:*** *A schematic overview of the generation of skin organoids using human induced pluripotent stem cells subjected to sequential treatment with different molecules. a) For the* *air-liquid interface culture using the transwell membrane organoids (T-SKO) group, on day 12 of differentiation, the organoids were transferred on a 6-transwell membrane (24 mm diameter, 0.4 µm pore size), where three organoids were transferred onto an individual well with 1.5ml of OMM, supplemented with 1% Matrigel. b) For the floating culture organoids (F-SKOs) group, on day 12 of differentiation, organoids were transferred into individual wells of a low attachment 24-well plate in 500μl of organoid maturation medium (OMM), supplemented with 1% Matrigel, and continued culturing in floating conditions. For floating organoids, on Day 15, 250μl of medium was removed from each well of the 24-well plate and replenished with 250μl of fresh OMM supplemented with 1% Matrigel. Full media change was performed for organoids in the transwell condition with fresh OMM supplemented with 1% Matrigel. Abbreviations: EB: embryoid body; CNC: cranial neural crest; E6: Essential 6 Medium; Y: Rho kinase inhibitor; SB: SB43152 TGF-β Receptor Inhibitor; FGF: Recombinant Human FGF-basic (FGF); BMP4: Recombinant Human BMP-4; LDN: Low dose naltrexone; OMM: organoid maturation medium; E8: Essential 8 Flex Medium.*


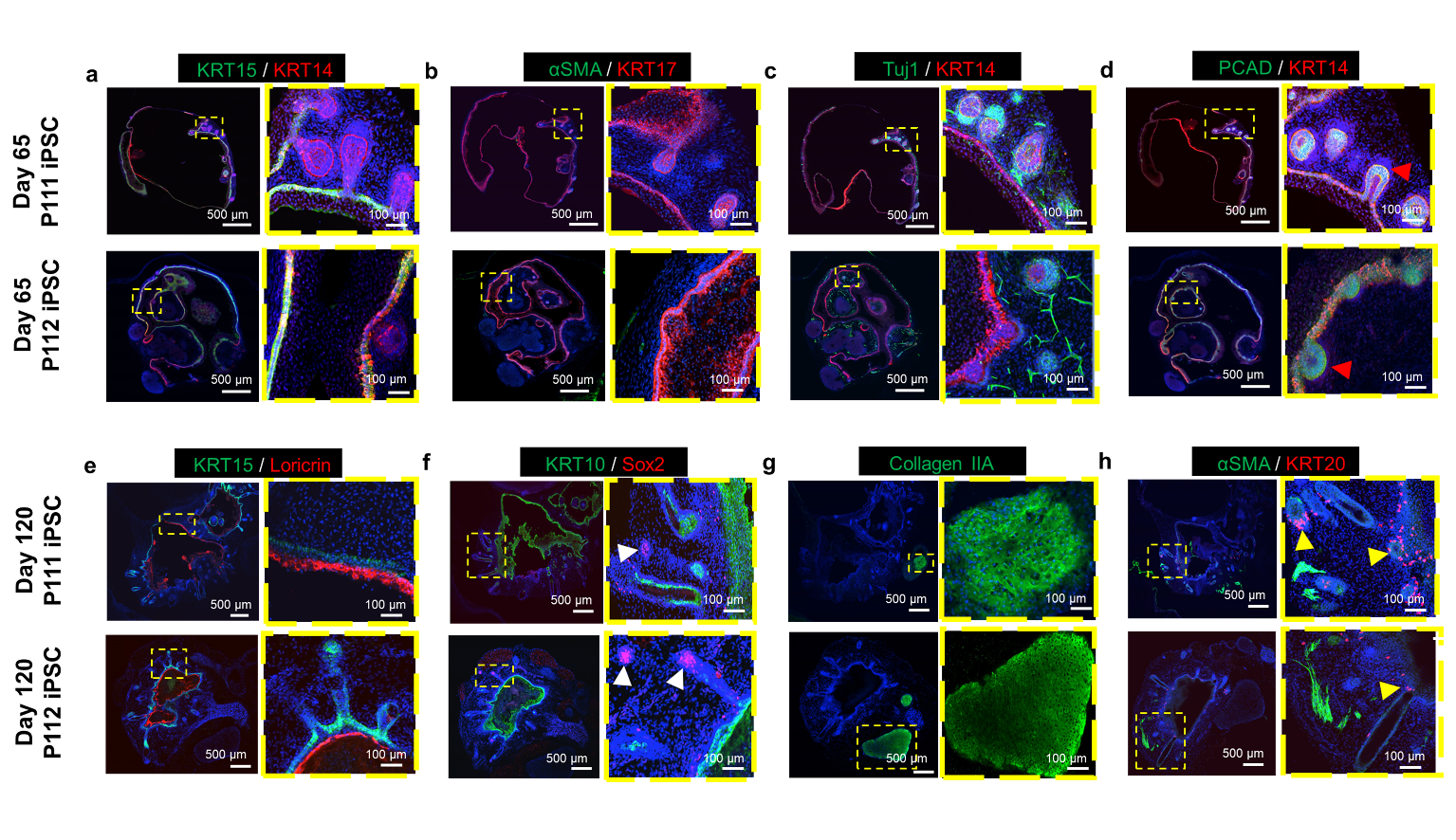


***Supplementary Figure 3:*** *Representative immunostaining images of P111 and P112 human induced pluripotent stem cell-derived skin organoids at Days 65 and 120 of differentiation using the air-liquid interface conditions. Red arrowheads in Part d represent the hair follicles (HFs). SRY-Box Transcription Factor 2 (Sox 2) staining in part f confirms the presence of dermal papilla cells at the HFs as indicated by the white arrowheads. The yellow arrowheads in part h show the Merkel cells. Blue indicates cell nuclei stained with 4′,6-diamidino-2-phenylindole (DAPI). Dashed yellow boxes indicate the magnified regions. Scale bars are 500 μm for the whole organoids and 100 μm for the magnified regions.*


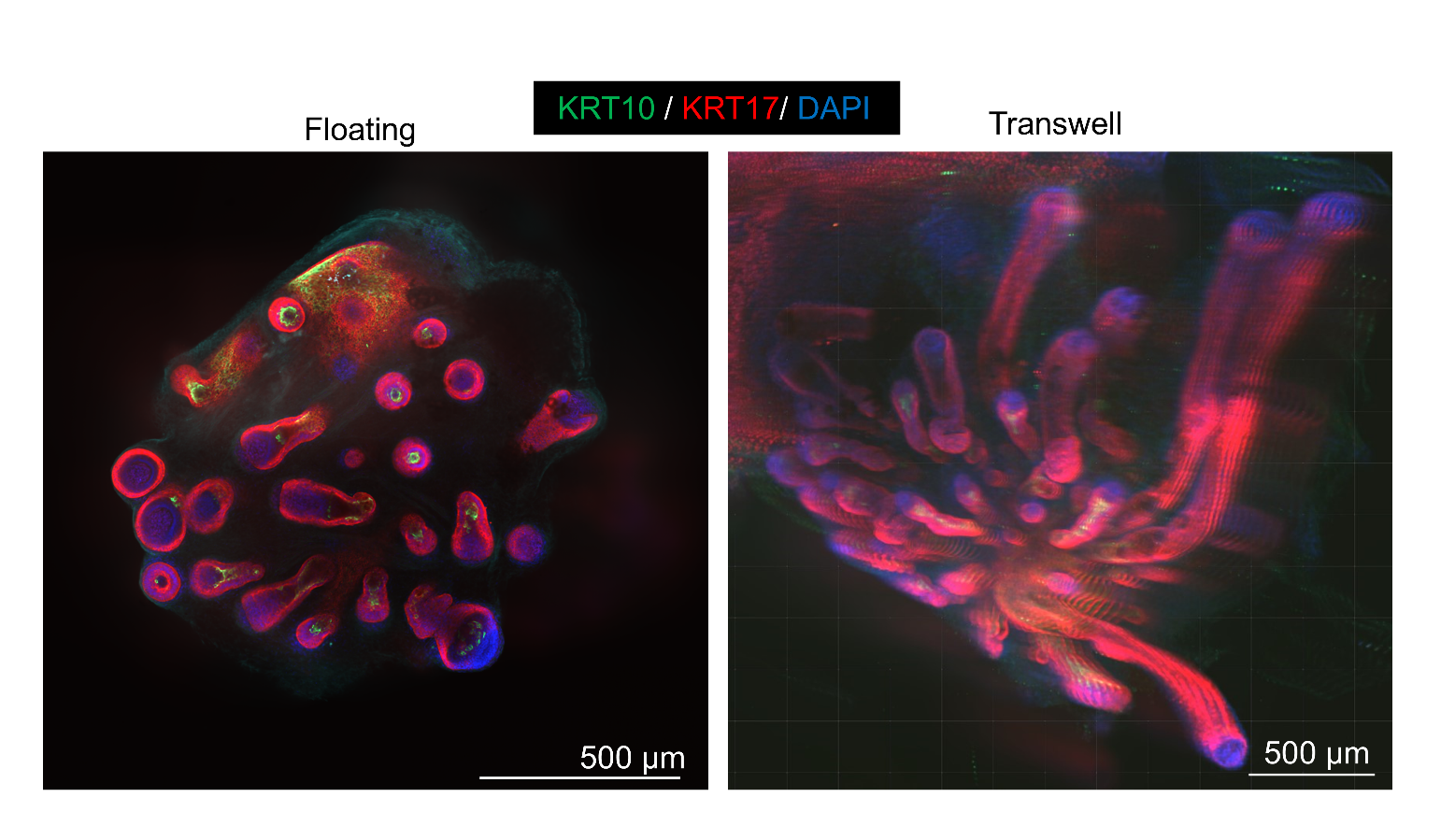


***Supplementary Figure 4:*** *Whole mount-immunostaining images of skin organoids (SKOs) differentiated using the floating and transwell conditions for 90 days and then immunostained with keratin 10 (KRT10, green color), and keratin 17 (KRT17, red color) antibodies. The SKOs exhibited longer hair follicles in the air-liquid interface conditions compared to those in floating conditions. Blue indicates cell nuclei stained with 4′,6-diamidino-2-phenylindole (DAPI). Scale bar: 500 µm.*

***Supplementary video 1:*** *A representative 3D reconstruction of skin organoids differentiated using the air-liquid interface conditions for 90 days and then immunostained with keratin 10 (KRT10, green color), and keratin 17 (KRT17, red color) antibodies. Scale bar: 300µm.*

***Supplementary video 2:*** *Representative 3D reconstructions of skin organoids differentiated using the air-liquid interface conditions for 120 days and then immunostained with keratin 10 (KRT10, green color), and keratin 17 (KRT17, red color) antibodies.*

***Supplementary video 3:*** *Representative 3D reconstruction of skin organoids differentiated using the air-liquid interface conditions for 130 days and then immunostained with keratin 10 (KRT10, green color), and keratin 17 (KRT17, red color) antibodies.*
